# Supplementary material for: Baseline haematological parameters in three common Australian frog species
Source: PeerJ. 2024 Jun 7;12:e17406. doi: 10.7717/peerj.17406 (PMC11164059; doi:10.7717/peerj.17406)
Supplement: Supplemental Information 3 [file peerj-12-17406-s003.docx]

MIQE Checklist relevant for the publication

Baseline haematological parameters in three common Australian frog species

Tara Jadwani-Bungar, Nicholas P Doidge, Danielle K Wallace, Laura A Brannelly*

| Item to check | Lines in the manuscript |
| --- | --- |
| *Sample*  Description  Volume processed  Processing procedure  Storage | 92-94  92-96  92-96  95, 102 |
| *Nucleic acid extraction*  Procedure  Name of kit and details of modifications  Source of additional reagents used | 96-101  96-97  98 |
| *qPCR oligonucleotides*  Primer sequence  Probe sequence  Manufacturer | 108-111  111-113  108-113 |
| *qPCR protocol*  Reaction conditions  Reaction volume  Primer and probe concentrations  Buffer identity and manufacturer  Additives  Thermocycling parameters  Manufacturer of qPCR instrument | 105-120  106  107-113  107  113  113-115 |
| *Data analysis*  qPCR analysis program  Results of NTCs  Outlier identification and disposition  Number of technical replicates  Statistical methods for results significance  Software | 122  118, 128-130  124-125  126-128  187-205  177 |
| *The qPCR performed in this study was for disease diagnostics therefore not all items on the MIQE checklist are relevant or performed. Here we only included the relevant items* | |
